# Sexual Risk Behaviors for HIV/AIDS in Chuuk State, Micronesia: The Case for HIV Prevention in Vulnerable Remote Populations

**DOI:** 10.1371/journal.pone.0001283

**Published:** 2007-12-12

**Authors:** Toya V. Russell, Ann N. Do, Eleanor Setik, Patrick S. Sullivan, Victoria D. Rayle, Carol A. Fridlund, Vu M. Quan, Andrew C. Voetsch, Patricia L. Fleming

**Affiliations:** 1 Office of Research, Uniformed Services University of the Health Sciences, Rockville, Maryland, United States of America; 2 Division of HIV/AIDS Prevention, National Center for HIV, STD, and TB Prevention, Centers for Disease Control and Prevention (CDC), Atlanta, Georgia, United States of America; 3 Chuuk State Department of Health and Hospital, Chuuk State, Federated States of Micronesia; 4 Global AIDS Program, National Center for HIV, STD, and TB Prevention, Centers for Disease Control and Prevention (CDC), Atlanta, Georgia, United States of America; 5 Department of Epidemiology, Johns Hopkins University, Bloomberg School of Public Health, Baltimore, Maryland, United States of America; 6 Department of Preventive Medicine and Community Health, New Jersey Medical School, University of Medicine and Dentistry of New Jersey, Newark, New Jersey, United States of America; Johns Hopkins Project, Malawi

## Abstract

**Background:**

After the first two cases of locally-acquired HIV infection were recognized in Chuuk State, Federated States of Micronesia (FSM), a public health response was initiated. The purpose of the response was to assess the need for HIV education and prevention services, to develop recommendations for controlling further spread of HIV in Chuuk, and to initiate some of the prevention measures.

**Methodology/Principal Findings:**

A public health team conducted a survey and rapid HIV testing among a sample of residents on the outer islands in Chuuk. Local public health officials conducted contact tracing and testing of sex partners of the two locally-acquired cases of HIV infection. A total of 333 persons completed the survey. The majority knew that HIV is transmitted through unprotected sexual contact (81%), injection drug use (61%), or blood transfusion (64%). Sexual activity in the past 12 months was reported among 159 participants, including 90 females and 69 males. Compared to women, men were more likely to have had multiple sex partners, to have been drunk during sex, but less likely to have used a condom in the past 12 months. The two men with locally acquired HIV infection had unprotected anal sex with a third Chuukese man who likely contracted HIV while outside of Chuuk. All 370 persons who received voluntary, confidential HIV counseling and testing had HIV negative test results.

**Conclusions/Significance:**

Despite the low HIV seroprevalence, risky sexual behaviors in this small isolated population raise concerns about the potential for rapid spread of HIV. The lack of knowledge about risks, along with stigmatizing attitudes towards persons infected with HIV and high risk sexual behaviors indicate the need for resources to be directed toward HIV prevention in Chuuk and on other Pacific Islands.

## Introduction

Although HIV has been recognized in populations on every continent of the world, few cases have been reported from the Pacific Islands – home to over 3.3 million people representing diverse indigenous cultures [1]; consequently, little attention has been paid to HIV prevention among the populations there [2]. However, these island populations remain particularly vulnerable to the spread of HIV because of their geographic location, poor health infrastructure, and high STD prevalence, reflecting risky sexual behaviors [3].

Based on experience in other parts of the world, an investment in behavioral risk reduction programs has tended to occur only after significant HIV-associated morbidity and mortality are evident [4]. However, the full benefit of HIV prevention is achievable only if the measures are implemented early, before the far-reaching consequences of a broader HIV epidemic come to pass. A coalition of advocates for the network of Pacific Islanders has been strident in calling for attention to the urgent preventive public health needs of these populations to forestall a repeat of the HIV crisis in some African and Asian countries.

In 2001, two cases of indigenous HIV transmission occurred in a small group of remote Pacific Islands outside of Weno, the capital of Chuuk State, Federated States of Micronesia (FSM). This group of islands is home to approximately 1500 residents and is reachable only by boat from the capital, Weno. Only limited primary health care is available through outreach to most of these outer island residents who otherwise must seek all health care at a single hospital located in Weno.

In response to the reports of locally-acquired HIV in Chuuk, the government of the Federated States of Micronesia (FSM) requested technical assistance from the CDC. Anecdotal reports of stigmatization and ostracization of two men with AIDS and their families suggested an urgent need for HIV education through community outreach. In addition, local health officials were conducting public health investigations involving the two men and their sexual contacts, and their preliminary findings indicated a need to provide voluntary HIV counseling and testing services to the residents of the group of outer islands.

Thus, the request for assistance among a remote Pacific Island population became an opportunity for public health officials to assess behavioral risks at the population level early (e.g., at the first sign of HIV's introduction), to determine prevention needs and identify public health interventions that could limit the spread of HIV. This manuscript describes the public health response to the first reports of locally acquired HIV infection in Chuuk. Our findings highlight the great need for and the special challenges of conducting HIV prevention activities in a limited-resource setting in the Pacific Islands, as well as contribute to current understanding about the HIV-related knowledge, attitude, and behaviors of individuals in this Micronesian population.

## Methods

### Overview

A CDC team joined a group of local public health officials in Weno in June 2001 to review the case investigation of two men with HIV infection who had resided in the remote outer islands of Chuuk. In addition to conducting a follow-up to the case investigation, the group of CDC and Chuukese public health professionals organized a survey to assess HIV knowledge, attitudes, and behaviors among the island residents. The group also provided interactive HIV educational sessions to the residents, distributed written HIV prevention materials and condoms, and conducted voluntary confidential HIV counseling and rapid testing. With the collaboration of public health officials in Weno, the HIV-related activities were integrated with other routine health outreach activities that included vaccinations, dental care, and mental health services; this was done to help enhance the acceptability of the HIV-related activities.

Upon arrival at each of the four outer islands, the local village officials convened a public meeting of the island residents to explain the purpose of general health outreach activities, including HIV counseling and testing services. Participants gave signed informed consent indicating their desire to receive confidential HIV counseling and/or to participate in the survey. Following the survey, an HIV information and health education session provided pre-test counseling. These sessions provided information about HIV transmission, prevention, treatment and voluntary testing. Eligible participants included adults who provided consent for themselves or children under 18 years of age who provided assent, and whose parents provided consent for their participation.

### Survey of knowledge, attitudes, and behaviors

A questionnaire was developed in English and translated into Chuukese. The survey was self-administered, with local health officials assisting respondents in interpreting the questionnaire if necessary. Because local cultural norms dictated that sensitive subjects such as sex behaviors are discussed with men and women separately, men and women were surveyed in separate locations. No link was kept between the consent forms and the anonymous survey responses. The questionnaire covered demographics, education, marital status, age at sexual initiation, sexual behaviors, self-report of symptoms suggestive of sexually transmitted infections, drug use, attitudes about HIV and knowledge of HIV risk factors. Data were entered at CDC and were analyzed using SAS version 8.2 (SAS Institute, Cary, NC, USA). We determined the frequencies of reported knowledge, attitudes, and behaviors concerning HIV. For those respondents who reported being sexually active in the prior twelve months, we assessed differences in the responses between men and women. Comparisons involving continuous variables were made by using the Wilcoxon rank-sum test, and those involving dichotomous variables were made by calculating odds ratios with 95% confidence intervals.

### Follow-up of two locally-acquired HIV/AIDS cases

Following the hospitalization in early 2001 of two men with diarrhea, dehydration and progressive weight loss who were diagnosed with HIV infection, local public health officials interviewed the men to identify their sexual contacts and to obtain a history of other HIV risk behaviors and potential exposures. There were no means to approach the named sexual contacts without risking the loss of confidentiality and attendant stigmatization and potential risk for violence. We therefore relied exclusively on the self-referral of contacts during a community-wide campaign of HIV counseling and testing events. Whenever known contacts presented for HIV counseling and testing, health department staff was able to notify these individuals of their potential exposure, encourage HIV testing, and provide expanded counseling on HIV risk reduction without loss of confidentiality. Following the campaign, the investigation remained open and local officials continued to attempt to identify named contacts presenting for voluntary HIV counseling and testing, to notify them of their potential exposure to HIV.

### HIV counseling and rapid testing

Due to the considerable logistical challenges of delivering confidential laboratory and diagnostic services in this remote island setting, the team offered rapid HIV testing to provide test results on the same day. Two rapid HIV tests, OraQuick (OraSure Technologies, Bethlehem, PA) and Determine (Abbott Laboratories, Abbott Park, IL), were conducted concurrently on the blood from each person tested. (Use of trade names is for identification only and does not imply endorsement by the Public Health Service or the U.S. Department of Health and Human Services.) If the results of the two concurrent tests were concordant, results were returned immediately (usually within 30 minutes of the fingerprick or venipuncture) [5]. Post-test counseling was provided individually at the time of the return of test results in makeshift rooms or outdoor areas where privacy could be afforded. For those with discordant test results, a dried blood spot on filter paper was sent for enzyme immunoassay (EIA) testing (conducted by Genetic Systems rLAV, Redmond, WA, approved 6/29/98 by U.S. Food and Drug Administration) and for Western blot (conducted at CDC in Atlanta, GA) testing as the “tie-breaker” test.

## Results

### Survey of knowledge, attitudes, and behaviors

A total of 333 persons participated in the survey of HIV-related risk behaviors, knowledge, and attitudes. Among these, 174 (52%) were female. The median age was 33 years (range 13 to 76 years). Among 269 participants who provided information on their educational level, 169 (63%) reported completing <12 years education.

Of the 333 participants, 218 (65%) reported that they had heard of HIV. Most (80%) were aware that HIV is incurable and can be spread through unprotected sex (81%), sharing of drug injection equipment (61%), or transfusion of infected blood (64%). However, only 31% indicated that they knew HIV can be transmitted from mother to child during birth. Some individuals also indicated that they thought HIV could be transmitted through mosquito bites (31%), kissing (23%), or handshaking (11%). Most participants reported being afraid of HIV-infected persons (72%), thought that HIV-infected persons “deserved it” (72%) or that HIV-infected persons should be jailed (58%).

Among the 333 participants, 224 (67%) reported ever having had sex. Among the 161 of these participants who specified the type of sex (vaginal, oral, and/or anal), 95% had had vaginal, 14% oral and 6% anal sex. A total of 159 participants (90 female and 69 male) reported they were sexually active in the past 12 months. Among these 159 individuals, the male participants, when compared to female participants, were younger, less likely to have had <12 years of education or to be married, and more likely to have initiated sex at a younger age and to have used injection drugs (Table 1). Regarding self-reported sexual behaviors in the previous 12 months, male participants were more likely than female participants to have had multiple partners, to have been drunk during sex, and were less likely to have used a condom during sex (Table 1).

**Table 1:** Characteristics of Male and Female Survey Participants Who Were Sexually Active in the 12 Months before an Anonymous Self-administered Survey, Chuuk, Federated States of Micronesia, June 2001.

Characteristics	Female Participants (n = 90)^a^	Male Participants (n = 69)^a^	Odds Ratio (95% CI)
Median age, years (range)^b^	35 (17–62)	24 (16–58)	_____
Highest educational level <12 years	56/81 (69%)	29/61 (48%)	2.5 (1.2–4.9)
Married	74/83 (89%)	31/63 (49%)	8.5 (3.6–19.8)
Median age at sexual initiation, years (range)^c^	19 (13–30)	16 (12–26)	_____
**Sexual behaviors in past 12 months:**			
Condom use during sex at least some of the time	68/74 (92%)	32/62 (52%)	10.6 (4.0–28.1)
Multiple (≥2) partners	14/90 (16%)	45/69 (65%)	0.1 (0.05–0.2)
Male-male sex	_____	11/69 (16%)	_____
Drunk during sex	9/72 (13%)	21/55 (38%)	0.2 (0.1–0.6)
Vaginal/urethral discharge in past 12 months	19/75 (25%)	15/48 (31%)	0.7 (0.3–1.7)
Knew where to obtain a condom	40/85 (47%)	23/57 (40%)	1.3 (0.7–2.6)
Afraid/embarrassed to obtain a condom	49/84 (58%)	24/54 (44%)	1.8 (0.9–3.5)
Ever injected drugs recreationally	1/76 (1%)	7/59 (12%)	0.1 (0.01–0.8)

a For each of the variables representing characteristics of male or female participants, any variation in the denominator is due to missing information (incomplete responses) among the total number (n) of individuals who participated in the survey.

b Information available for 86 female and 66 male participants; p<0.01, Wilcoxon rank-sum test.

c Information available for 74 female and 59 male participants; p<0.01, Wilcoxon rank-sum test.

Among individuals who were sexually active in the previous 12 months, there were several self-reported behaviors which may indicate high risk for HIV infection. Male-to-male sex was reported by 11 (16%) of the 69 men surveyed. Sixty (38%) – including 45 men and 15 women – had multiple (≥2) sex partners, and 25 (42%) of the 60 were married. Among the 45 men with multiple partners, 9 (20%) were men who had sex with men, and 8 of these 9 men also reported having had female partners in the past 12 months. Most (63%) individuals with multiple sex partners reported some condom use [endorsed using condoms either “every time”, “most of the time”, or “some of the time”] during sex in the previous 12 months. Although 58% of men with multiple partners reported condom use during sex at least “some of the time,” only 9% reported condom use “most of the time,” and 4% reported condom use “every time.” Among 15 women with multiple partners, only 4 (29%) reported condom use “most of the time,” and none reported condom use “every time.”

### Follow-up of two locally-acquired HIV/AIDS cases

The first locally-acquired HIV/AIDS cases were recognized among two young men, both of whom had multiple male and female sex partners. Both men reported having had unprotected anal intercourse in Chuuk with a common male sex partner (hereafter called the index case) who later died from AIDS-related illnesses. The index case was a native Chuukese who had lived abroad before he became ill, was diagnosed with HIV infection, and returned to Chuuk. While living abroad, the index case had multiple male sex partners and used injection drugs.

The two locally-infected men reported 31 other sex partners (28 male and 3 female partners), in addition to the index case described above. Of these 31 partners, 15 (including the two wives of the two young men) received voluntary HIV counseling and testing; none was found to be seropositive for HIV at the time of testing. The remaining 16 sex partners were not notified of their potential HIV exposure during the initial wave of HIV outreach efforts because these individuals did not seek HIV counseling and testing services during that time, and there were no feasible means to initiate contact with them in a confidential manner on the islands (e.g., no telephone, no private mailing address). Local officials have continued to work to confidentially contact these additional sex partners who may be at risk for HIV infection and to offer voluntary counseling and testing to them.

### HIV counseling and rapid testing

A total of 370 individuals received pre-test counseling, gave consent and provided specimens for HIV testing using the Determine and Oraquick rapid tests. Of these, 368 specimens had negative results on both rapid tests. Two other blood specimens had discordant results on the rapid tests, but both specimens showed negative HIV-1 Western Blot results, and were thus considered to be overall negative tests. Of the 370 individuals who were tested for HIV, 357 (96%) returned for post-test counseling.

## Discussion

With the introduction of HIV into the remote outer islands of Chuuk, our findings regarding the practice of risky sexual behaviors among the island residents has raised some concerns about the potential for further spread of HIV with possible devastating consequences, as has been observed in other developing countries [6]. In our survey, the lack of knowledge about HIV risks and stigmatizing attitudes toward persons infected with HIV highlighted the challenges of HIV prevention and control, calling attention to the importance of assessing and addressing the HIV prevention needs of population outer islands in Chuuk and, perhaps, of other similar remote Pacific Island populations as well.

Based on our observations, persons at greatest risk for HIV infection among the survey participants were young men, particularly a small group of men who have sex with men (MSM). The prevalence of male-male sex in our survey was higher than has been reported in national probability surveys in the United States and the United Kingdom [7]. Locally-acquired cases of HIV infection were first recognized among two MSM who reported having had sex with many male partners. Among the participants in our survey, more men than women reported having had multiple sex partners, and about 20% of the male participants who were sexually active had same-sex partners in the past 12 months. Overall, a lower proportion of men than women reported any condom use during sex, and among men with multiple sex partners, only 58% reported any condom use during sex in the past 12 months. Younger men, in particular, may be at risk for HIV infection, because men reported sexual initiation at a younger age than women.

Nevertheless, the HIV risk to Chuukese women is also of concern. Sixteen percent of women reported having had multiple sex partners in the previous 12 months, and the rate of consistent condom use among this small sample of women was low. Even women with one sex partner may have a substantial HIV risk because 18% of the men with multiple sex partners reported having both male and female sex partners. In addition, knowledge about the perinatal transmission of HIV is lacking, indicating a need for further education on this issue among women.

Both male and female outer island residents are affected by the lack of public health resources in Chuuk. While most of our survey participants understood that HIV can be transmitted through unprotected sex, many, including those with multiple partners, did not use a condom during sex. Not all who were surveyed knew where they could obtain condoms. Some of the participants had confidentiality concerns about asking for condoms in a public setting. Structural barriers were also important in restricting access to condoms – which are not readily available in the small remote outer islands. Although there was not a specific question in our survey regarding transportation, it was clear that transportation from the outer islands to the public health clinic on Weno was difficult, depending on the availability of a functioning boat and adequate fuel, as well as safe travel conditions at sea. In addition, the material resources to purchase condoms are not always accessible to those living in the outer islands. These factors are also indicative of limitations in other important resources for basic medical care, including HIV counseling and testing services and screening and treatment for sexually transmitted diseases.

Consequently, outreach activities may be critical to the overall HIV prevention efforts in Chuuk. An important part of our outreach activities included the use of HIV rapid testing, which allowed results to be returned within the same day and may reduce problems with failure to return for test results [8]. The availability of rapid HIV testing was especially valuable in a setting such as the outer islands in Chuuk, where geographic barriers and logistic difficulties restrict not only the access to testing services but also the adequate storage and timely transport of specimens to a laboratory for EIA and western blot testing. Rapid testing is recommended by the World Health Organization and the CDC for use in settings where EIA is not practical or where there is limited laboratory infrastructure [5], and rapid testing has been approved by the FDA for use in the U.S. since 2002 [9]. Testing algorithms using combinations of two or more rapid tests, similar to that used in our survey, have been demonstrated to be reliable, with sensitivity and specificity comparable to those of traditional EIA and western blot testing [7], [10]–[11]. Additional HIV prevention outreach efforts have occurred in Chuuk since 2001, including HIV rapid testing and prevention and education messages based in part on the results of our survey.

The data from our survey have important limitations. We were not able to assess the representativeness of our data due to the lack of population census information. Our surveys and other outreach activities appeared to be well-received by those who participated, but there may be self-selection bias, since individuals participated voluntarily. Given the sensitive nature of questions in the surveys, there also may have been some underreporting of risk behaviors. The prevalence of anal sex was consistent with other surveys, but the prevalence of oral sex was markedly lower [7].

Based on our findings in Chuuk, we recommend continued outreach efforts to disseminate factual information about HIV and reduce stigmatization, to increase the availability and promote the use of condoms, to provide on-site HIV counseling and testing services, and to conduct ongoing data collection to assess HIV-related knowledge, attitudes, and behaviors. We emphasize the concern that geographic isolation and poverty complicate the provision and sustainability of these basic public health services for these island populations. It is crucial that ongoing resources and attention be given to HIV monitoring and prevention efforts in Chuuk and in the surrounding Pacific Island area, before the full impact of a local HIV/AIDS epidemic devastate these historically important and culturally rich island populations, as it has in Africa and in other developing areas of the world.
